# Supplementary material for: Tryparedoxin peroxidase-deficiency commits trypanosomes to ferroptosis-type cell death
Source: eLife. 2018 Jul 26;7:e37503. doi: 10.7554/eLife.37503 (PMC6117152; doi:10.7554/eLife.37503)
Supplement: Supplementary file 1. — (A) Bloodstream T. brucei were cultured for 1, 2 or 3 days in the presence of 100 µM Trolox, 200 nM Liproxstatin-1 or 100 nM Ferrostatin-1 as well as different RSL3 concentrations and then subjected to plate reader-based ATPlite measurements. Chlorhexidine, a trypanocidal compound and known inhibitor of trypanothione reductase (TR) (Meiering et al., 2005; Beig et al., 2015), served as positive control. 1The data are the mean of at least two independent series of experiments each conducted in triplicate with standard deviations (SD). 2The values are the mean of an experiment conducted in triplicate with SD. (B) NADPH, T(SH)2, TR, and Tpx ± Px were incubated with 40 µM RSL3. After different times, the assays were started by adding (a) 100 µM H2O2 or (b) Px and H2O2. The data were derived from at least double determinations which varied by ≤ 10%. [file elife-37503-supp1.docx]

**Tryparedoxin peroxidase-deficiency commits trypanosomes to ferroptosis-type cell death**

**Marta Bogacz and R. Luise Krauth-Siegel**

**Supplementary file 1**

**A.** Trypanocidal activity of RSL3 towards bloodstream *T. brucei*

EC_50_-value ± SD (µM)

Treatment 24 h 48 h 72 h

(*1S,3R*)-RSL3^1^ 2.1 ± 1.1 1.9 ± 0.6 4.5 ± 1.8

RSL3 racemate^1^ 3.6 ± 1.2 3.7 ± 1.5 8.1 ± 1.9

RSL3 racemate + Trolox^1^ 2.2 ± 0.8 2.4 ± 0.6 7.6 ± 2.3

(*1S,3R*)-RSL3 + Trolox^2^ 2.3 ± 0.3 3.5 ± 0.0 8.7 ± 0.3

(*1S,3R*)-RSL3 + Liproxstatin-1^2^ 3.1 ± 0.2 3.6 ± 0.0 8.6 ± 0.2

(*1S,3R*)-RSL3 + Ferrostatin-1^2^ 2.3 ± 0.3 3.7 ± 0.2 8.3 ± 0.6

Chlorhexidine^1^ 0.5 ± 0.1 0.3 ± 0.1 0.4 ± 0.2

**B.**  *In vitro* inhibitory potency of RSL3 on the parasite peroxidase cascade

Inactivation (%)

Incubation time (min) + Px^a^ - Px^b^

1 20 17

15 26 19

40 40 39
